# Supplementary material for: Nonredox trivalent nickel catalyzing nucleophilic electrooxidation of organics
Source: Nat Commun. 2023 Dec 2;14:7987. doi: 10.1038/s41467-023-43649-6 (PMC10693638; doi:10.1038/s41467-023-43649-6)
Supplement: Supplementary file 1 — Supplementary Information [file 41467_2023_43649_MOESM1_ESM.pdf]

Nonredox trivalent nickel catalyzing nucleophilic electrooxidation of organics

Yuandong Yan[1], Ruyi Wang[1], Qian Zheng[1], Jiaying Zhong[1], Weichang Hao[2], Shicheng Yan[1,3*], and Zhigang Zou[1,3]

[1]Collaborative Innovation Center of Advanced Microstructures, National Laboratory of Solid State Microstructures, College of Engineering and Applied Sciences, Nanjing University, No. 22 Hankou Road, Nanjing, Jiangsu 210093, P. R. China.

[2]School of Physics, Beihang University, 37 Xueyuan Road, Beijing, 100191 P. R. China

[3]Jiangsu Key Laboratory for Nano Technology, Eco-materials and Renewable Energy Research Center (ERERC), Nanjing University, No. 22 Hankou Road, Nanjing, Jiangsu 210093, P. R. China.

*Correspondence: **yscfei@nju.edu.cn**

Table of Contents

Supplementary Figures 1-21
Supplementary References

Supplementary Figures

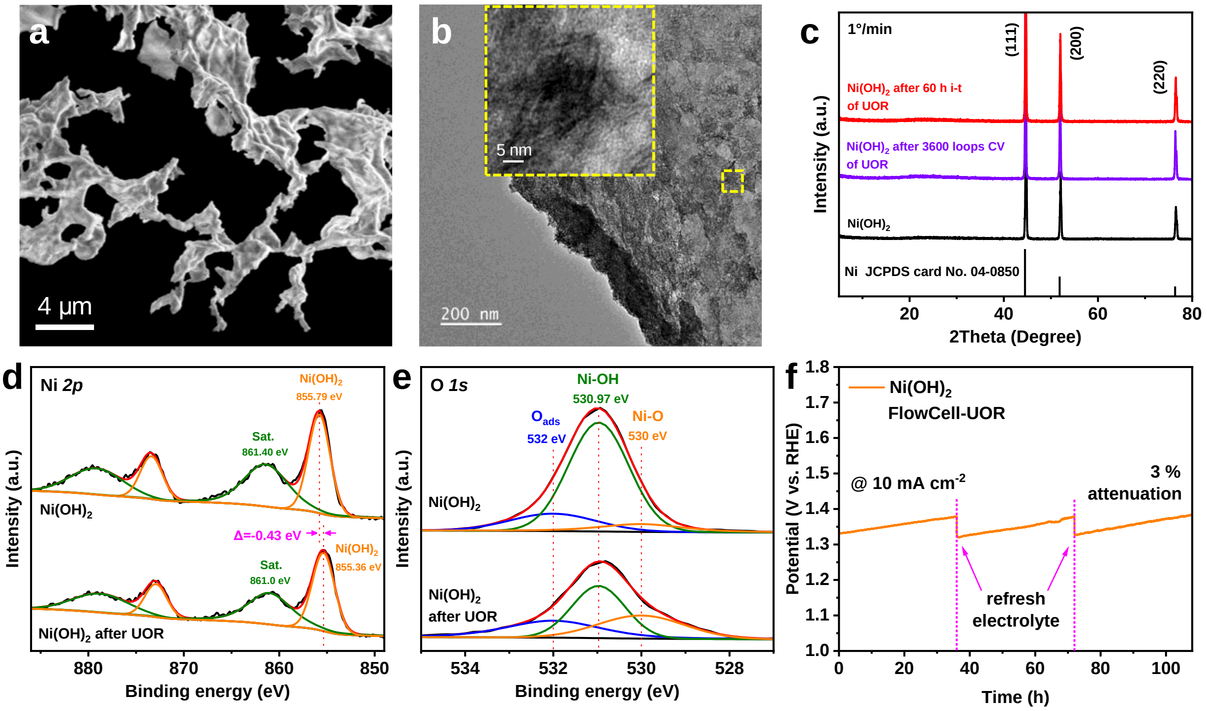

Supplementary Figure 1 | $Ni(OH)_2$ electrode. ***a*****,** Scanning electron microscopy (SEM) image of $Ni(OH)_2$ electrode. ***b*****,** Transmission electron microscopy (TEM) image of $Ni(OH)_2$ electrode. The Inset is a high-resolution TEM lattice image for an area as indicated by the yellow dotted box. ***c*****,** XRD patterns for the as-prepared $Ni(OH)_2$ electrode, the $Ni(OH)_2$ electrode after 3600 CV scans in 0.33 M urea + 1 M KOH electrolyte, and the $Ni(OH)_2$ electrode after i-t for 60 h in 0.33 M urea + 1 M KOH electrolyte. ***d*****,** Ni *2p* XPS spectra for the as-prepared $Ni(OH)_2$ and the $Ni(OH)_2$ after UOR. ***e*****,** O *1s* XPS spectra for the as-prepared $Ni(OH)_2$ and the $Ni(OH)_2$ after UOR. ***f*****,** Stability in 10 mA cm^{-2} current density for $Ni(OH)_2$ electrode for over 110 h. The dotted lines represent the replacement of fresh 0.33 M urea + 1 M KOH electrolyte.

The SEM image shows that the electrodeposited catalyst is a $Ni(OH)_2$ gel (Supplementary Fig. 1a). The XRD patterns indicated that the $Ni(OH)_2$ catalyst is amorphous for the as-prepared electrode, the electrode after 3600 CV scans or i-t test for 60 h in 0.33 M urea + 1 M KOH electrolyte (Supplementary Fig. 1b). The XPS analysis confirmed that the amorphous $Ni(OH)_2$ is stable during UOR (Supplementary Figs. 1d and e). The high UOR stability suggests that the amorphous $Ni(OH)_2$ is an ideal model for the study of UOR on Ni-based catalysts (Supplementary Fig. 1f).

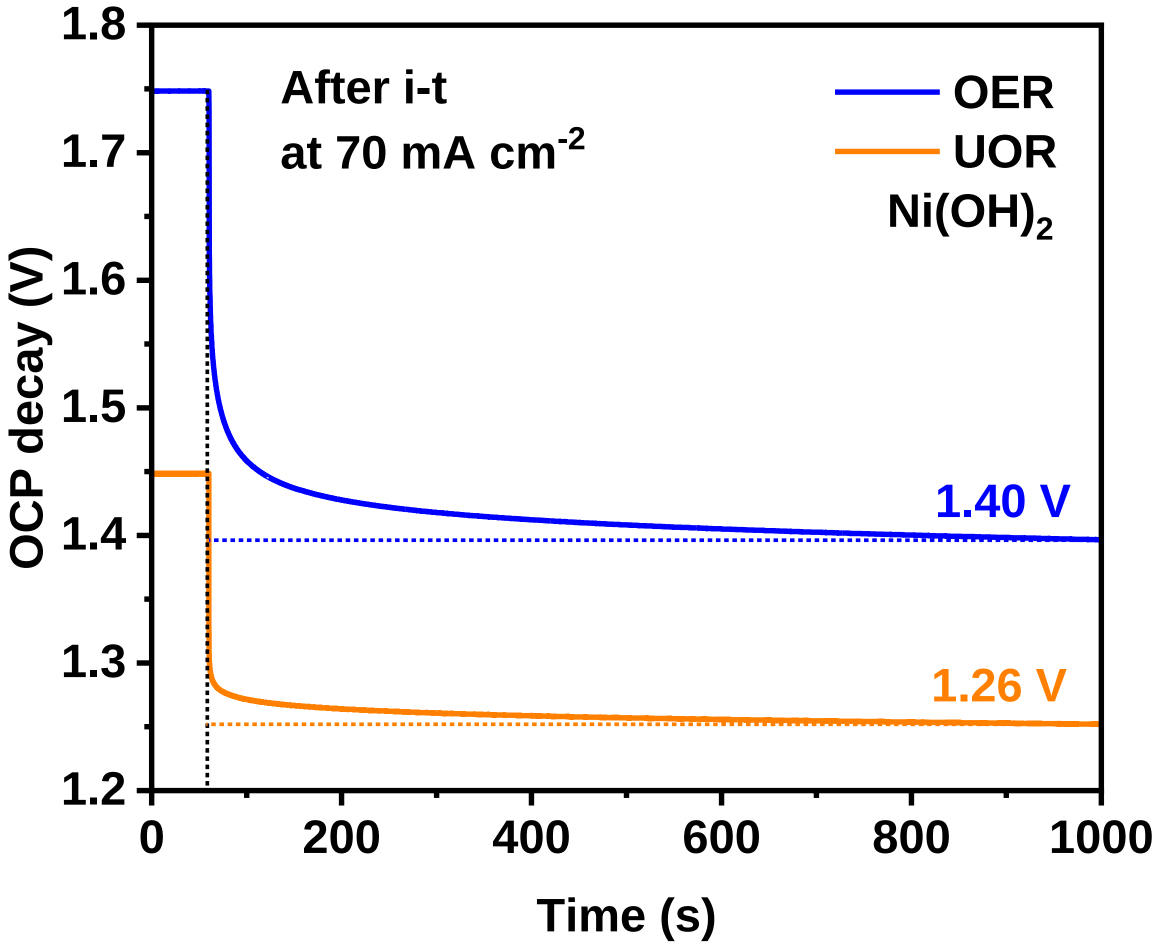

Supplementary Figure 2 | Open-circuit potential (V_{OCP}) decay after polarizing the $Ni(OH)_2$ electrode at 70 mA cm^{-2} in 1 M KOH electrolyte or 0.33 M urea + 1 M KOH electrolyte.

After polarizing the $Ni(OH)_2$ electrode at 70 mA cm^{-2}, the V_{OCP} decay in 1 M KOH electrolyte stopped at a quasi-equilibrium potential of 1.40 V, a potential for Ni^{3+} generation. And the V_{OCP} decay in 0.33 M urea + 1 M KOH electrolyte stopped at a quasi-equilibrium potential of 1.26 V, a potential for Ni^{2+} generation. This result demonstrated that the Ni^{3+} is not capable of oxidizing H_2O and works for urea oxidation.

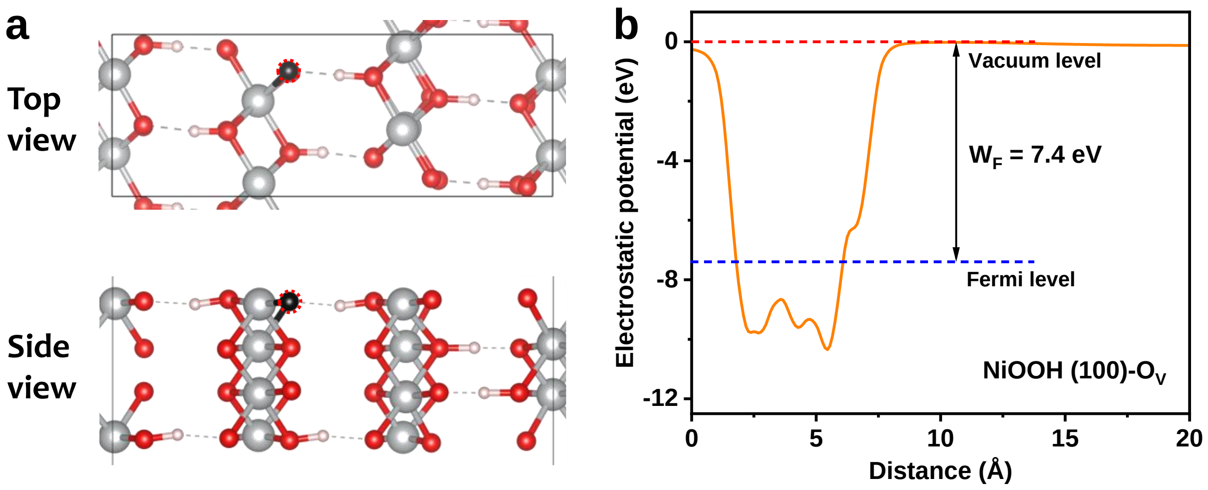

Supplementary Figure 3 | Fermi level calculation of NiOOH (100)-O_V. a, Structure model of NiOOH (100), in which the silver, red, black, and white spheres are nickel, oxygen, oxygen vacancy, and hydrogen, respectively. And the oxygen atom to be removed to form NiOOH (100)-O_V was marked by the red dashed circle. **b,** The work function of NiOOH (100)-O_V.

The Fermi level (E_F) for a given solid model is defined by the equation $E_F = E_{VAC} - W_F$, where vacuum level (E_{VAC}) is the energy level of an electron at rest far away from any solid surface, and work function (W_F) is the energy barrier to free space that prevents an electron at the Fermi level from escaping the solid [1]. The calculated E_F of NiOOH (100)-O_V is -7.4 eV.

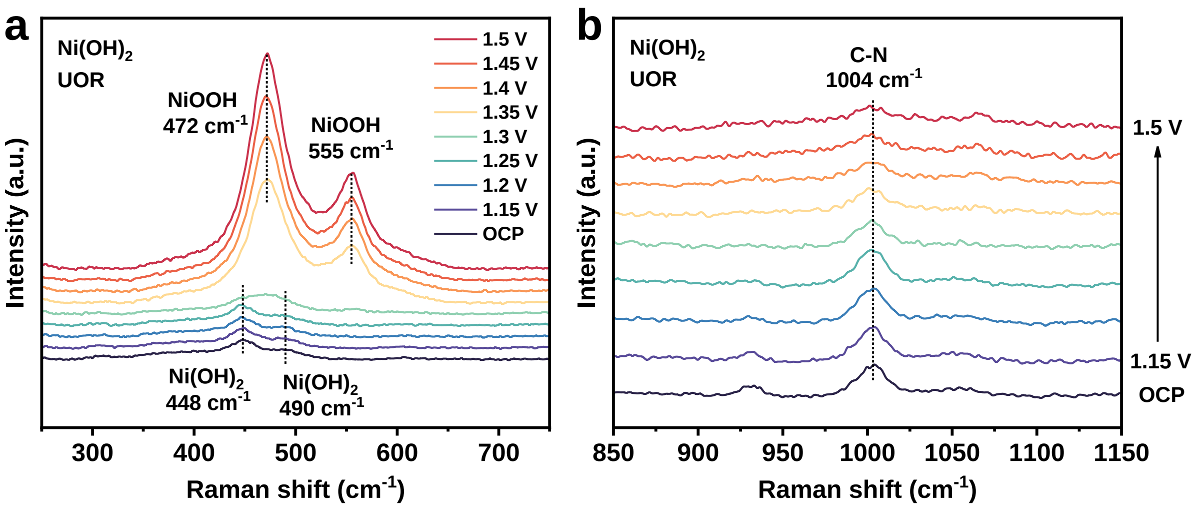

Supplementary Figure 4 | In situ Raman spectra of $Ni(OH)_2$ electrode during UOR at **a,** 250-750 cm^{-1}, and **b,** 850-1150 cm^{-1}.

As shown in Supplementary Fig. 4a, polarizing the electrode in 0.33 M urea + 1 M KOH electrolyte, the in situ Raman bands at 448 cm^{-1} and 490 cm^{-1} from V_{OCP} to 1.25 V are attributed to bending vibration of E_g (δ(Ni-O)) and stretching vibration of A_{1g} (ν(Ni-O)) of $Ni(OH)_2$. When potentials above 1.35 V, the new bands at 472 cm^{-1} and 555 cm^{-1} are assigned to bending vibration of E_g (δ(Ni-O)) and stretching vibration of A_{1g} (ν(Ni-O)) of NiOOH. Meanwhile, the C-N signal of urea at 1004 cm^{-1} decreased from 1.35 to 1.5 V (Supplementary Fig. 4b), indicating the consumption of urea.

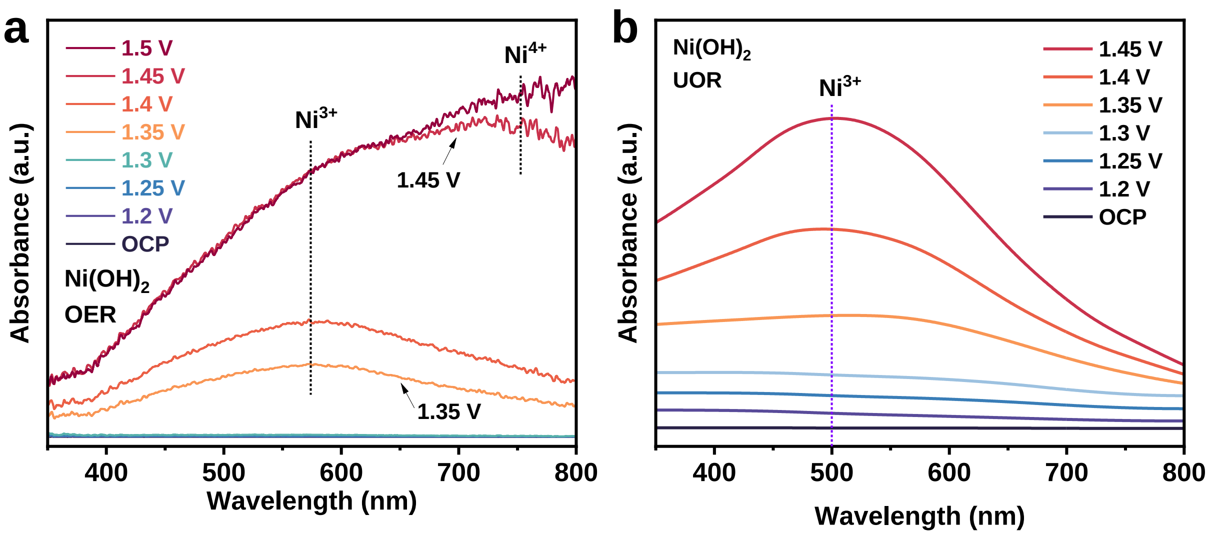

Supplementary Figure 5 | In situ UV–vis absorption spectra of $Ni(OH)_2$ electrode during **a,** OER and **b,** UOR. The peak at 570 nm occurring at potentials above 1.35 V is attributed to Ni^{3+} generation during OER. And the absorption peak of Ni^{3+} is observed at 500 nm when potentials are above 1.3 V.

As shown in Supplementary Fig. 5a, the absorption of Ni^{3+} at 570 nm was observed during OER by in-situ electrochemical UV-Vis absorption spectrum at potentials above 1.35 V in 1 M KOH electrolyte [2]. As the applied potentials increase above 1.45 V, absorption peak with a wavelength longer than 750 nm appears, corresponding to the generation of high-valence Ni^{4+} species, indicating that water is oxidized by Ni^{4+} species. However, the absorption of Ni^{3+} occurs at 500 nm during UOR at potentials above 1.35 V in 0.33 M urea + 1 M KOH electrolyte (Supplementary Fig. 5b). The absorption peak red-shift for Ni^{3+} would indicate the different electronic states for active Ni^{3+} during OER and UOR. Indeed, the high barriers for Ni^{3+} reacting with H_2O make the Ni^{3+} stay at a resting state in 1 M KOH and the Ni^{3+} works in the urea solution to transfer electrons with a strong interaction with the urea.

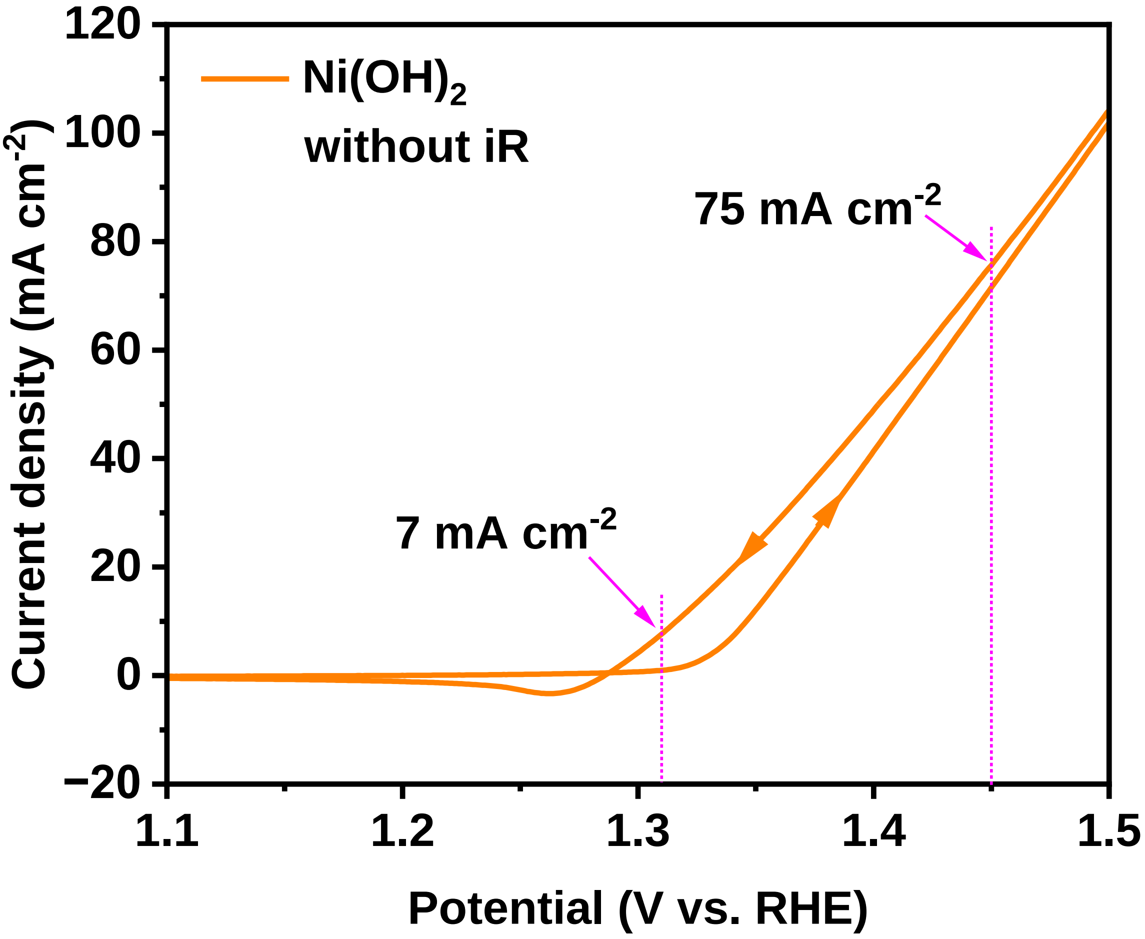

Supplementary Figure 6 | CV curve of $Ni(OH)_2$ electrode in 0.33 M urea + 1 M KOH electrolyte at a scan rate of 5 mV s^{-1} without iR compensation. The i-t test (Fig. 3f) and CV scan at a scan rate of 5 mV s^{-1} (Supplementary Fig. 6) exhibit the same current density at 1.31 and 1.45 V, revealing the extremely rapid electron transfer to create new equilibrium state during UOR.

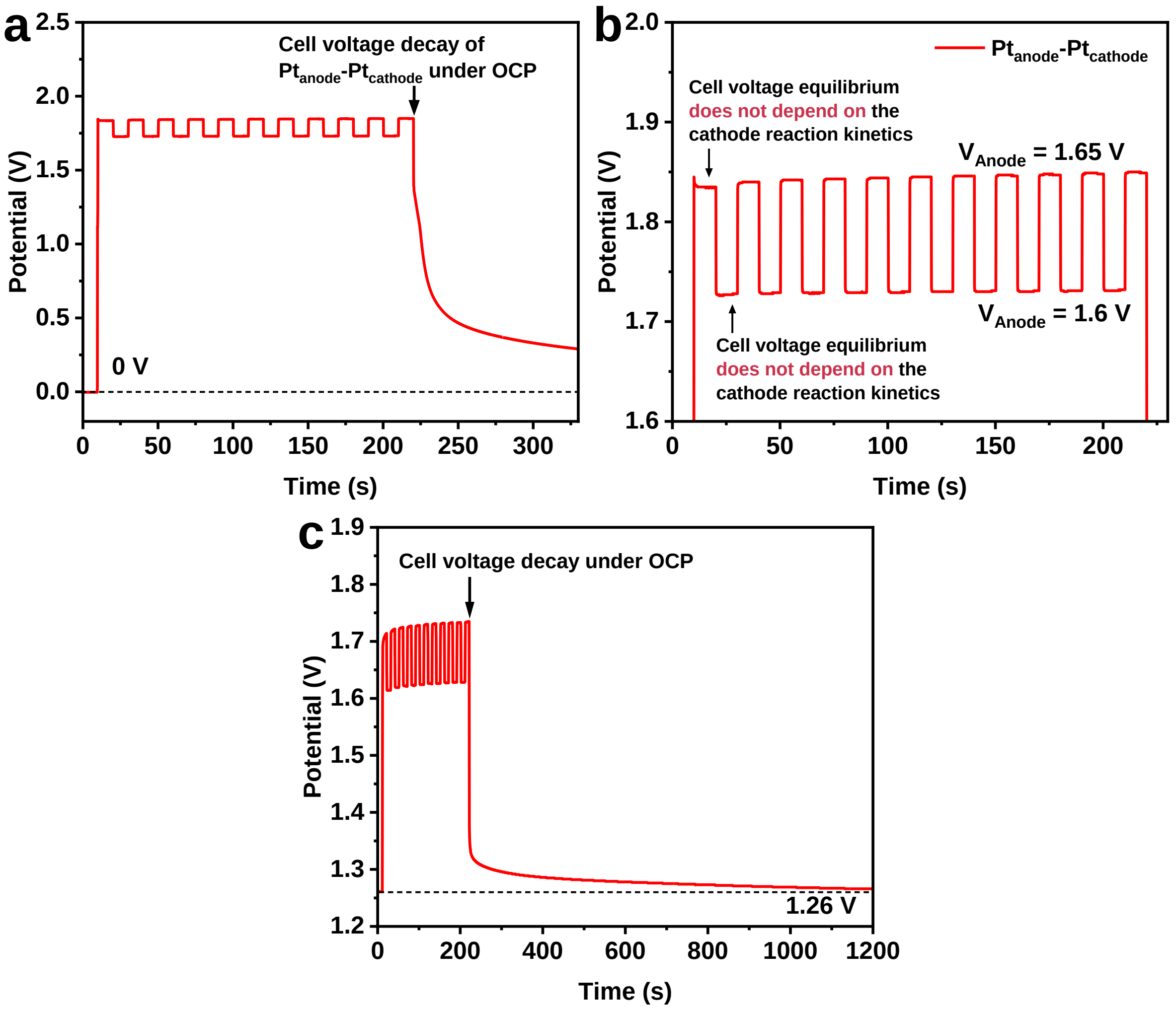

Supplementary Figure 7 | a, Cell voltage decay of Pt$_{anode}$-Pt$_{cathode}$ after periodically altering the anodic potentials between 1.6 and 1.65 V with a stay time of 10 s at every potential point. **b,** The time-dependent cell voltage equilibrium of Pt$_{anode}$-Pt$_{cathode}$ when periodically altering the anodic potentials between 1.6 and 1.65 V. **c,** Cell voltage decay after periodically altering the anodic potentials between 1.4 and 1.45 V with a stay time of 10 s at every potential point. Under open-circuit conditions, the cell voltage is decayed to 1.26 V, which is the same to the quasi-equilibrium potential for the decay of anodic open-circuit potential (Fig. 3e).

Abruptly altering the anodic potentials between 1.6 and 1.65 V on the Pt foil, the cell voltage immediately reaches the equilibrium state, indicating fast transient kinetics on both cathode of Pt foil and anode of Pt foil, to rapidly adjust the Fermi level of the electrode, thus sensitively adapting to the potential changes. These facts confirmed that the cell voltage is mainly dominated by the anodic potentials.

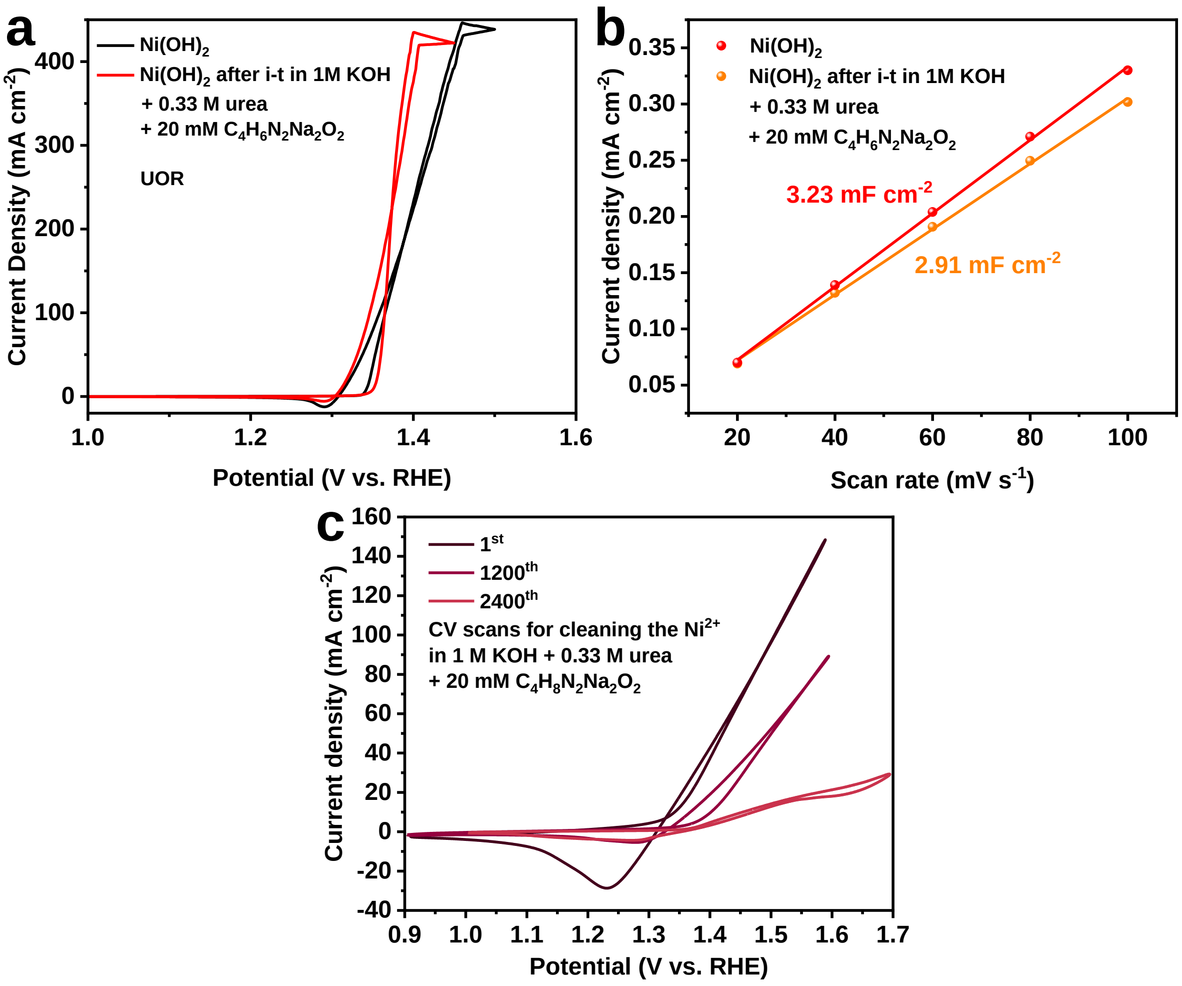

Supplementary Figure 8 | a, CV polarization curves for as-prepared $Ni(OH)_2$ electrode and the $Ni(OH)_2$ electrode after at initial 1.55 V for 6 h and subsequent 1.31 V for 24 h in 20 mM $C_4H_8N_2Na_2O_2$ + 0.33 M urea + 1 M KOH. **b,** The linear relationship between the current density and scan rate of $Ni(OH)_2$ electrode, which is used to calculate the electrochemical active surface area (ECSA). The CV scans were carried out in a non-faradaic potential region from 0.95 to 1.05 V. The current density at 1.0 V was used for the calculation of the ECSA. The electrical double-layer capacitance (C_{dl}) was calculated by the equation $C_{dl} = (j_a\text{-}j_c)/2\nu$, where j_a and j_c are the anodic current density and cathodic current density, respectively, *and* ν is the scan rate. Thus, C_{dl} is the slope of the linear relationship between $(j_a\text{-}j_c)/2$ and scan rates. The ECSA can be calculated by ECSA = $C_{dl}/C_{dl,\ Ref}$, where $C_{dl,\ Ref}$ is the specific capacitance of an ideal flat surface of the catalyst. Here, we use the average value of 0.04 mF cm^{-2} for $C_{dl,\ Ref}$ in alkaline solutions without taking the used material and measurement conditions into account. **c,** CV curves of $Ni(OH)_2$ electrode without iR compensation for cleaning the Ni^{2+} in 20 mM $C_4H_8N_2Na_2O_2$ + 0.33 M urea + 1 M KOH for 2400 loops.

No remarkable changes in LSV and electrical double-layer capacitance were visible before and after constant voltage testing at initial 1.55 V for 6 h and subsequent 1.31 V for 24 h in 20 mM $C_4H_8N_2Na_2O_2$ + 0.33 M urea + 1 M KOH, strongly suggesting that no surface Ni^{2+} species on the electrode was stripped during UOR. This fact is a solid evidence to demonstrate that the urea oxidation by Ni^{3+} does not undergo the $Ni(OH)_2$/NiOOH redox couple to transfer electrons.

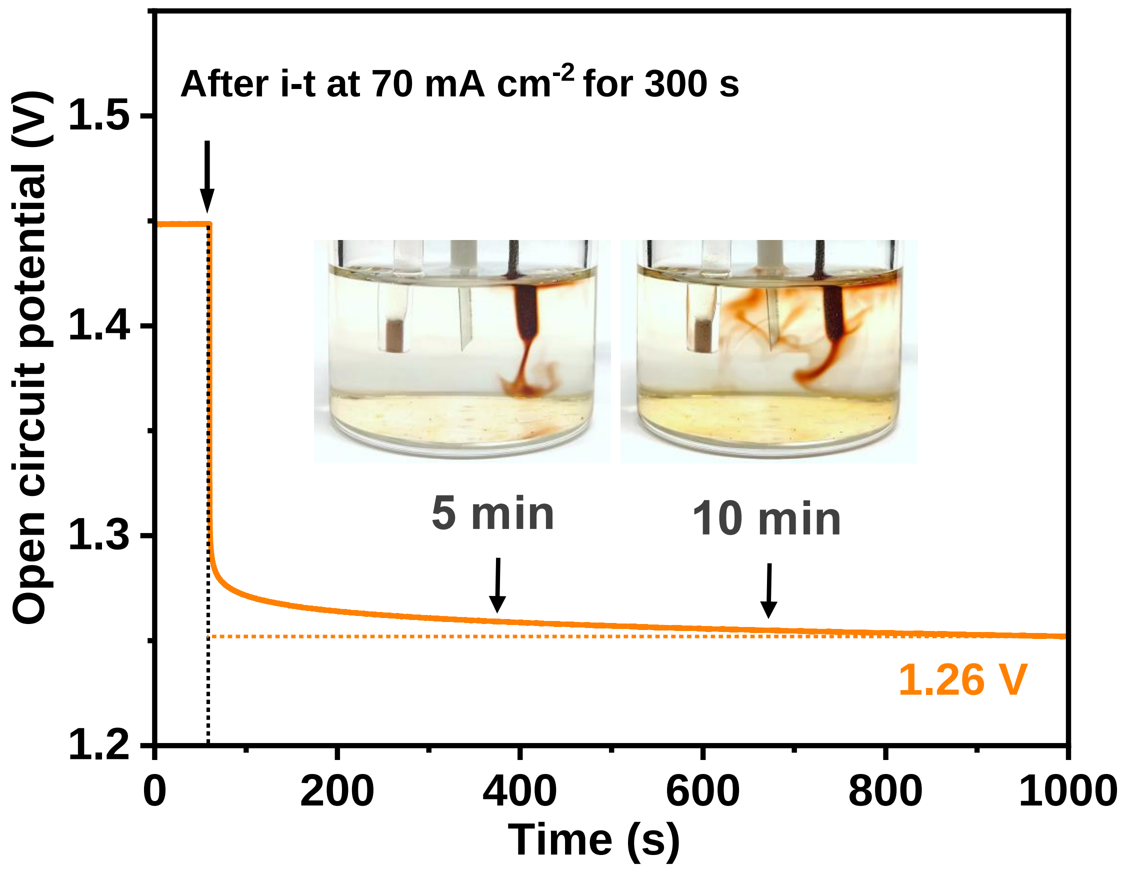

Supplementary Figure 9 | The V_{OCP} decay after polarizing $Ni(OH)_2$ at 70 mA cm^{-2} in 20 mM $C_4H_8N_2Na_2O_2$ + 0.33 M urea + 1 M KOH. Insets show the optical photos of electrolytes to show the color changes of electrolytes under open circuit conditions for 5 min and 10 min.

As shown in Supplementary Fig. 9, the complexing reaction was confirmed to occur after spontaneous chemical reduction of Ni^{3+} by urea under V_{OCP} conditions, as indicated by color changes of wine-red soluble complexes at 5 min and 10 min. This evidence well rules out a possibility of transferring electrons by $Ni(OH)_2$/NiOOH redox couple during UOR. The activity of Ni^{3+} is significant enough to chemically oxidize urea, making us believe that the applied potentials above $Ni(OH)_2$/NiOOH oxidation potential are mainly responsive for extracting electrons that are received from urea into Ni^{3+}.

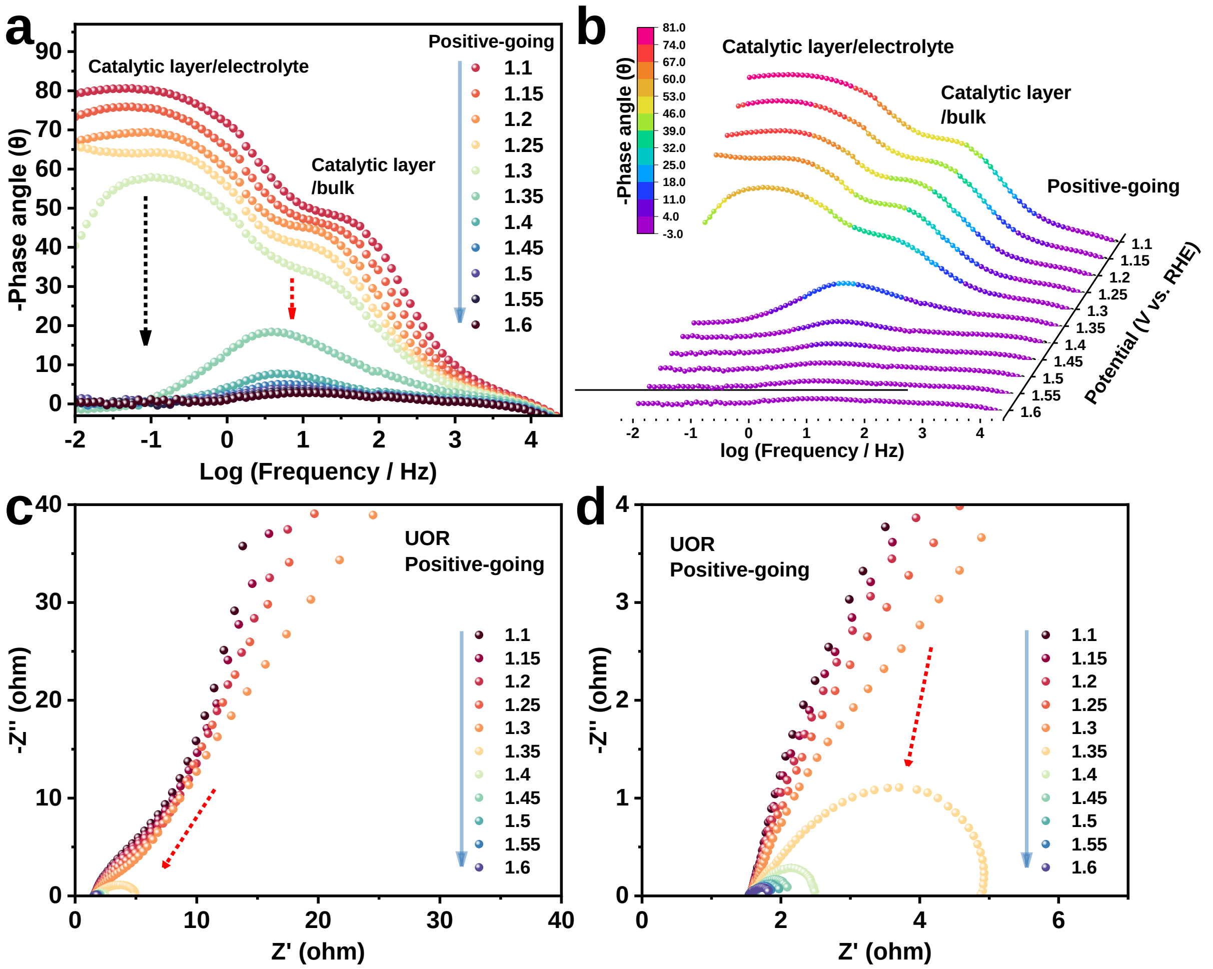

Supplementary Figure 10 | Potential-dependent EIS Nyquist and Bode plots of $Ni(OH)_2$ electrode when potentials positive-going varied from 1.1 to 1.6 V in 0.33 M urea + 1 M KOH electrolyte. **a,** Potential-dependent Bode plots shown in phase angle-logarithmic frequency coordinate system. **b,** Bode plots shown in phase angle-logarithmic frequency-potentials coordinate system. **c,** Potential-dependent Nyquist plots. **d,** The potential-dependent Nyquist plots in the low-frequency region of Supplementary Fig. 10c.

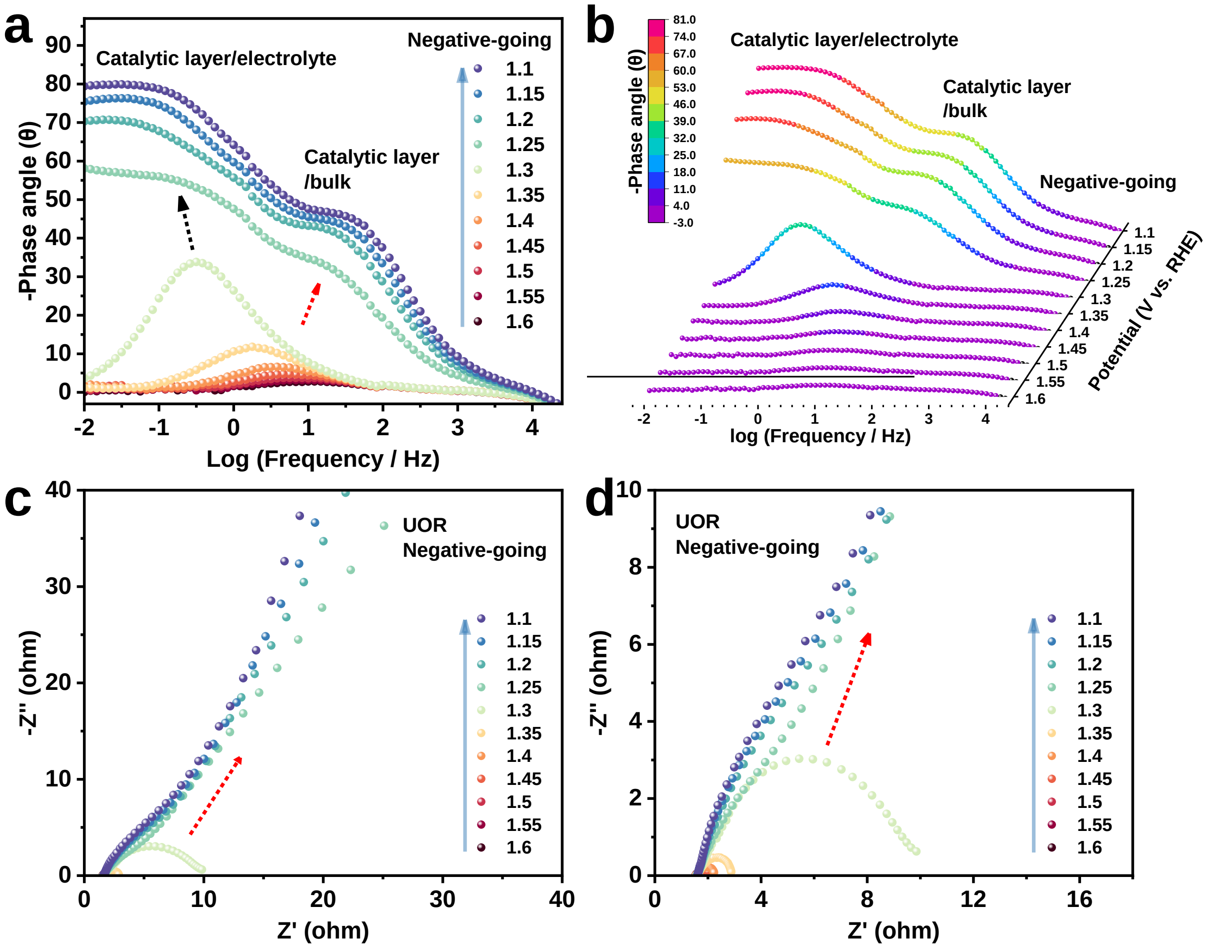

Supplementary Figure 11 | Potential-dependent EIS Nyquist and Bode plots of $Ni(OH)_2$ electrode when potentials negative-going varied from 1.6 to 1.1 V in 0.33 M urea + 1 M KOH electrolyte. Potential-dependent Bode plots shown in phase angle-logarithmic frequency coordinate system. **b,** Bode plots shown in phase angle-logarithmic frequency-potentials coordinate system. **c,** Potential-dependent Nyquist plots. **d,** The Potential-dependent Nyquist plots in the low-frequency region of Supplementary Fig. 11c.

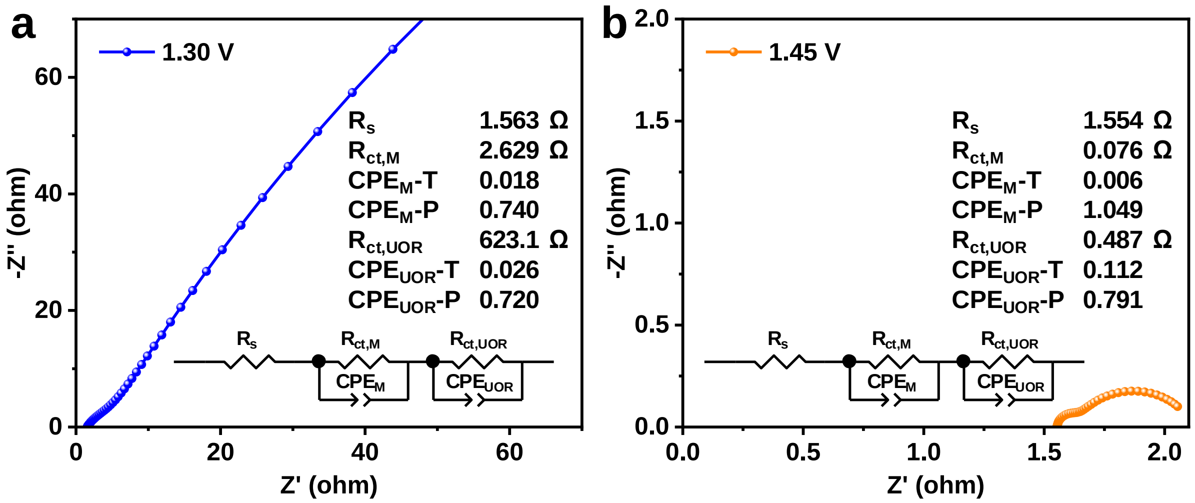

Supplementary Figure 12 | Fitting analysis of EIS Nyquist plots. The data were from Supplementary Fig. 10 for $Ni(OH)_2$ electrode at ***a,*** 1.30 V and ***b,*** 1.45 V. The insets show the equivalent circuit and the fitting data.

We fitted the Nyquist plot by a typical Randle's circuit, which was composed of solution and catalyst-conductive substrate electrical connection resistance (R_s), electron transfer resistance, and constant phase angle element to describe the capacitance for the catalyst bulk/catalytic layer interface ($R_{ct,M}$, CPE_M-T, and CPE_M-P) and the catalytic layer/electrolyte interface ($R_{ct,UOR}$, CPE_{UOR}-T, and CPE_{UOR}-P), as shown in Supplementary Fig. 12. When the applied potential is higher than 1.30 V, a UOR potential, both the $R_{ct,M}$ and $R_{ct,UOR}$ are as low as 0.076 - 0.487 ohm, suggesting that the electron transfer during UOR on NiOOH is similar to the electron conduction in a metallic conductor.

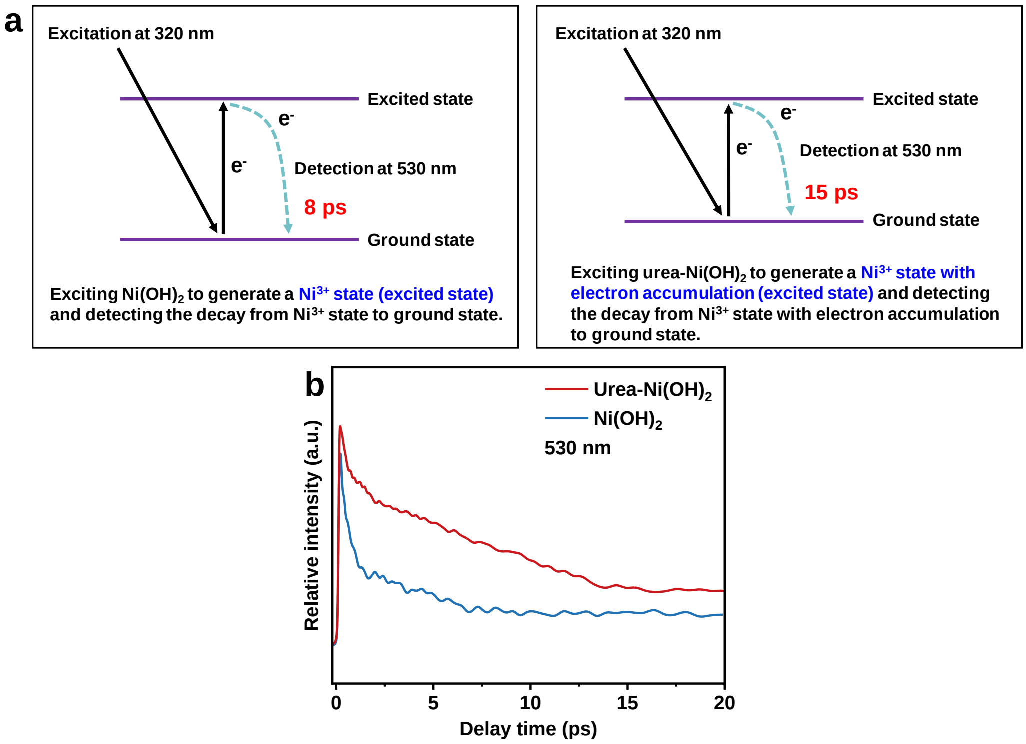

Supplementary Figure 13 | Transient absorption spectra to check the electron transfer kinetics in Ni^{3+} state and Ni^{3+} state with electron accumulation. a, Schematic diagram of excited state and ground state. The adsorption of urea tends to stabilize the $Ni(OH)_2$ at low valence due to that the urea molecule with nucleophilic carbonyls is an electron donor. Accordingly, the adsorption of urea is likely to charge the surface of $Ni(OH)_2$ to form surface-charged $Ni(OH)_2$, thus slightly increasing the ground state of $Ni(OH)_2$. Therefore, the Ni in $Ni(OH)_2$ and urea-$Ni(OH)_2$ can be considered to be Ni^{3+} state and Ni^{3+} state with electron accumulation, respectively. That is, the decay of excited-state $Ni(OH)_2$ and excited-state urea-$Ni(OH)_2$ are able to reflect the electron transfer kinetics in Ni^{3+} state and Ni^{3+} state with electron accumulation, respectively. **b,** Femtosecond transient absorption spectra of $Ni(OH)_2$ and urea-$Ni(OH)_2$ detected by 530 nm probe light after excitation at 320 nm pump light.

To provide an experimental evidence to show the electron transfer kinetics in the Ni^{3+} state and Ni^{3+} state with electron accumulation in a picosecond or femtosecond time scale, the femtosecond transient absorption spectra (fs-TAS) were carried out. According to the XPS results (Supplementary Fig. 1d), the adsorption of urea tends to stabilize the $Ni(OH)_2$ at low valence due to that the urea molecule with nucleophilic carbonyls is an electron donor. This means that the strong interactions between urea and $Ni(OH)_2$ change the surface electronic states of $Ni(OH)_2$. Obviously, the Ni species on the surface of urea-$Ni(OH)_2$ represent a lower valence state compared to Ni species on the surface of $Ni(OH)_2$. Accordingly, the adsorption of urea is likely to charge the surface of $Ni(OH)_2$ to form surface-charged $Ni(OH)_2$, thus slightly increasing the ground state of $Ni(OH)_2$. We found that the $Ni(OH)_2$ can be excited by 320 nm pump light and the light absorption of the excited-state $Ni(OH)_2$ is similar to that of Ni^{3+} with a characteristic absorption at 500 nm - 550 nm. As shown in Supplementary Fig. 13a, after light excitation at 320 nm, both the $Ni(OH)_2$ and urea-$Ni(OH)_2$ are excited from ground state to an excited state. Therefore, the Ni in excited-state $Ni(OH)_2$ and excited-state urea-$Ni(OH)_2$ can be considered to be Ni^{3+} state and Ni^{3+} state with electron accumulation, respectively. That is, the decay of the excited-state $Ni(OH)_2$ and urea-$Ni(OH)_2$ is able to reflect the electron transfer kinetics in Ni^{3+} state and Ni^{3+} state with electron accumulation, respectively. We detected the decay kinetics from the exited state to ground state at 530 nm. As shown in Supplementary Fig. 13b, the decay time for the excited-state $Ni(OH)_2$ and urea-$Ni(OH)_2$ is 8 ps and 15 ps, respectively, indicating the charge transfer on Ni^{3+} state was faster than that on the Ni^{3+} state with charge accumulation. Thus, electrons tend to transfer through Ni^{3+} state directly, rather than accumulating to form transient Ni^{2+} state which hinders electron transfer.

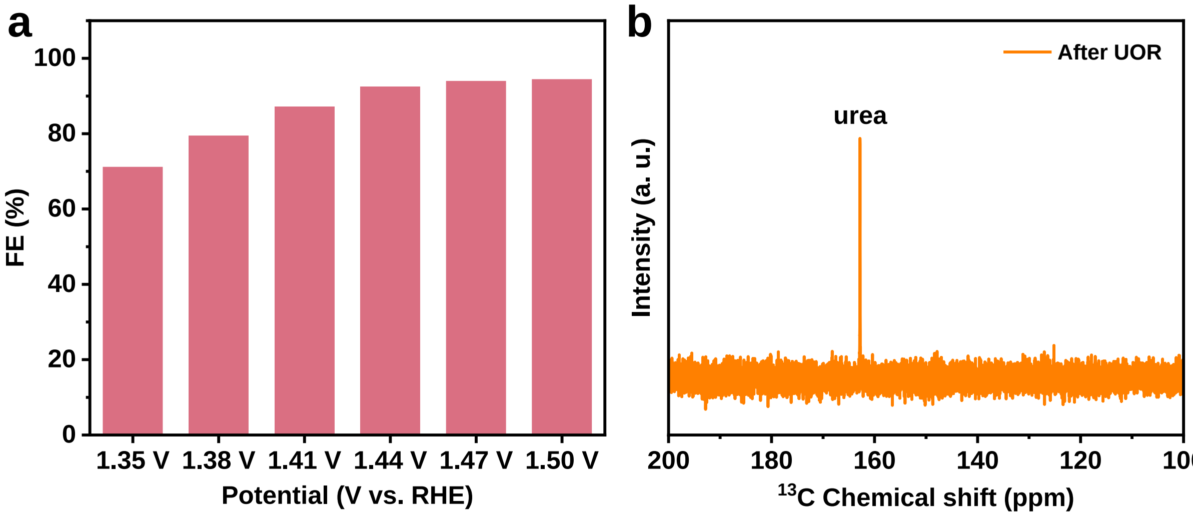

Supplementary Figure 14 | a, The N_2 faradaic efficiency at anodic potentials between 1.35 and 1.5 V. **b,** The ^{13}C NMR spectra to identify the UOR product.

To confirm that the UOR on $Ni(OH)_2$ electrode is a catalytic reaction, we have checked the product quantification, selectivity, and faradaic efficiency. The electrooxidation of urea follows a reaction of $CO(NH_2)_2 + 6OH^- \rightarrow N_2 + CO_2 + 5H_2O + 6e^-$. This means that the UOR products are the gaseous N_2 and CO_2 and liquid H_2O. Considering that both the H_2O and CO_2 are soluble in 1 M KOH, the product, N_2, was analyzed by gas chromatography (GC) to confirm the selectivity and faradaic efficiency of UOR. The Faraday efficiency for N_2 is 94.5 % (near 100%, the deviation mainly resulted from the N_2 dissolving in electrolyte) at potentials above 1.4 V as shown in Supplementary Fig. 14a. The electron transfer amounts for N_2 generation were equal to the amount of electric charge that passed through the electrode during UOR, suggesting a UOR catalytic reaction to occur. Furthermore, the selectivity of UOR was confirmed by the ^{13}C NMR spectra to detect the liquid products of long-time UOR. As shown in Supplementary Fig. 14b, no liquid products were detected after UOR, suggesting the high product selectivity for overall urea oxidation to CO_2, N_2, and H_2O.

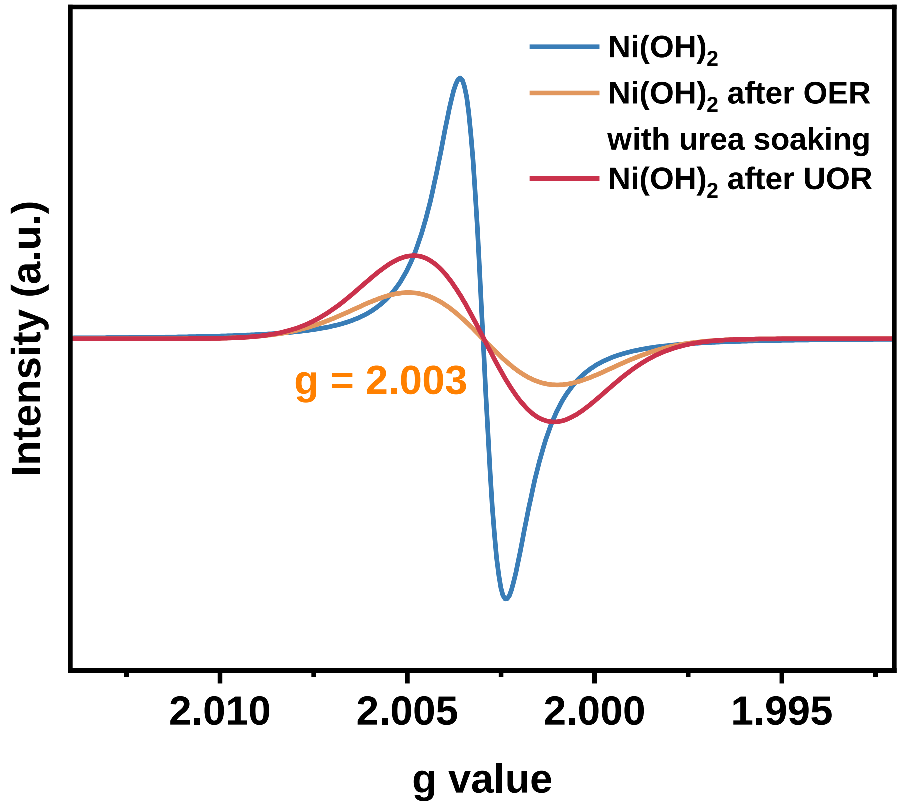

Supplementary Figure 15 | EPR signals for the as-prepared $Ni(OH)_2$ electrode, the $Ni(OH)_2$ electrode after UOR, and the $Ni(OH)_2$ electrode after OER with urea soaking.

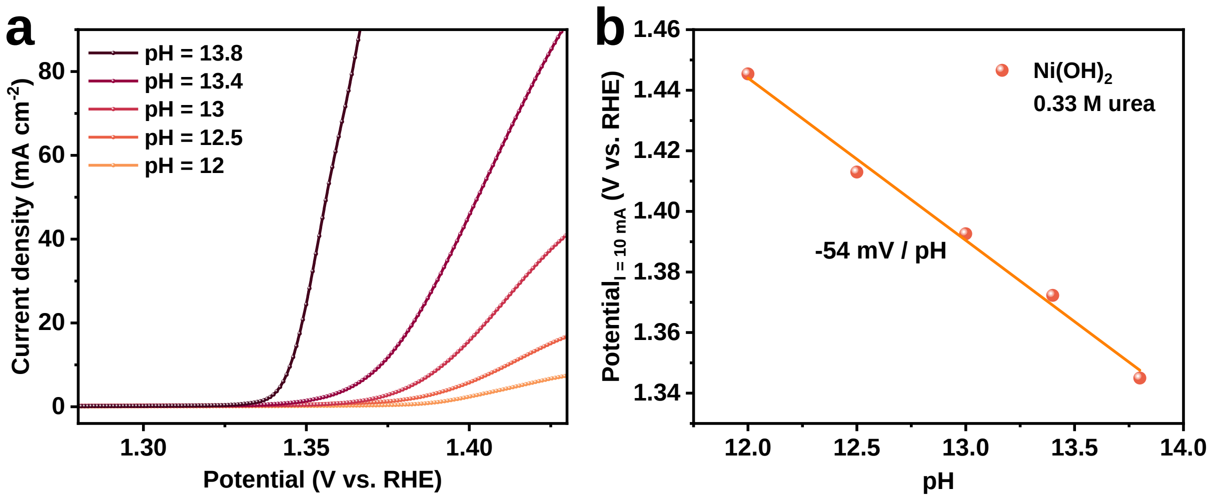

Supplementary Figure 16 | a, LSV curves for $Ni(OH)_2$ electrode in 0.33 M urea-containing electrolyte with different pH values. To avoid the conductivity difference caused by the ion concentration difference, K^+ concentration was balanced to 1 M with the addition of K_2SO_4. **b,** The linear relationship of potential at 10 mA current vs. pH for $Ni(OH)_2$ electrode.

Nernst equation under standard temperature and pressure (25 °C, 1 atm), $\varphi = \varphi^0 + \frac{RT}{nF}\sum v_i \ln a_i$, where φ is equilibrium potential, φ^0 is theoretical equilibrium potential, R is gas constant, T is absolute temperature, n is electron transfer number of reaction, F is faraday constant, v_i is solvated stoichiometric number of reactive substance, negative for the reduction state and positive for the oxidation state, a_i is activity of the reactive substance, is used to predict the equilibrium potential of the electrochemical reaction. The chemical equation of anodic reaction of UOR is $CO(NH_2)_2 + 6OH^- - 6e^- = N_2 + CO_2 + 5H_2O$. In theory, if the protons in the UOR are highly solvated to be released into the electrolyte, $v_{OH^-} = 6$ and $n = 6$. According to the Nernst equation, the equilibrium condition for this reaction under 25 °C and 1 atm is

$$\begin{aligned}\varphi &= \varphi^0 + \frac{RT}{nF}\ln\frac{a_{N_2}a_{CO_2}a_{H_2O}^5}{a_{CO(NH_2)_2}a_{OH^-}^6}\\ &= \varphi^0 - \frac{RT}{nF}\ln a_{OH^-}^6\\ &= \varphi^0 - \frac{2.3RT}{6F} * 6 * \log a_{OH^-}\\ &= \varphi^0 - 0.059pH\end{aligned}$$

The pH dependence of UOR potential-current curves was recorded to understand the UOR mechanism (Supplementary Fig. 16a). To exclude the influence of background current and $Ni(OH)_2/NiOOH$ current, we obtain the initial UOR potential by the threshold current density method [3] to define a potential at 10 mA cm^{-2} as UOR potential ($V_{10\ mA}$). Here, the $V_{10\ mA}$ of UOR on $Ni(OH)_2$ electrode is a linear function of pH with a negative slope of -54 mV pH^{-1} (Supplementary Fig. 16b), much close to -59 mV pH^{-1} slope of theoretical $V_{10\ mA}$ *versus* pH for UOR with the completely solvated protons. Thus, the hydrogen of urea is released to electrolyte by the solvated reaction during UOR.

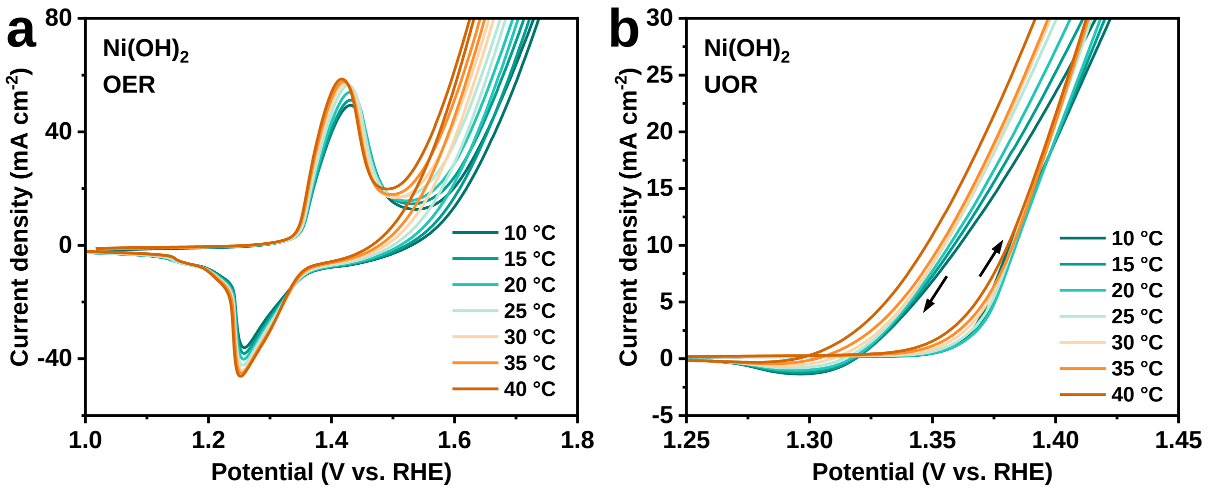

Supplementary Figure 17 | Temperature-dependent polarization curves at a scan rate of 5 mV s^{-1} in **a,** 1 M KOH, and **b,** 0.33 M urea + 1 M KOH.

The activation energy for electrooxidation of $Ni(OH)_2$ to NiOOH ($Q_{Ni^{2+/3+}}$), electroreduction of NiOOH to $Ni(OH)_2$ ($Q_{Ni^{3+/2+}}$), and reduction of NiOOH by urea to $Ni(OH)_2$ ($Q_{Ni^{3+/2+}, urea}$) were calculated by Arrhenius plot of inverse temperature versus the log of $Ni(OH)_2$/NiOOH oxidation peak current, Ni^{3+}/Ni^{2+} reduction peak current, and Ni^{3+}/Ni^{2+} reduction peak current in 0.33 M urea + 1 M KOH, respectively. The activation energy for UOR (Q_{UOR}) was calculated by Arrhenius plot of inverse temperature versus the log of UOR current density at potential which is $Ni(OH)_2$/NiOOH oxidation peak potential in 1 M KOH.

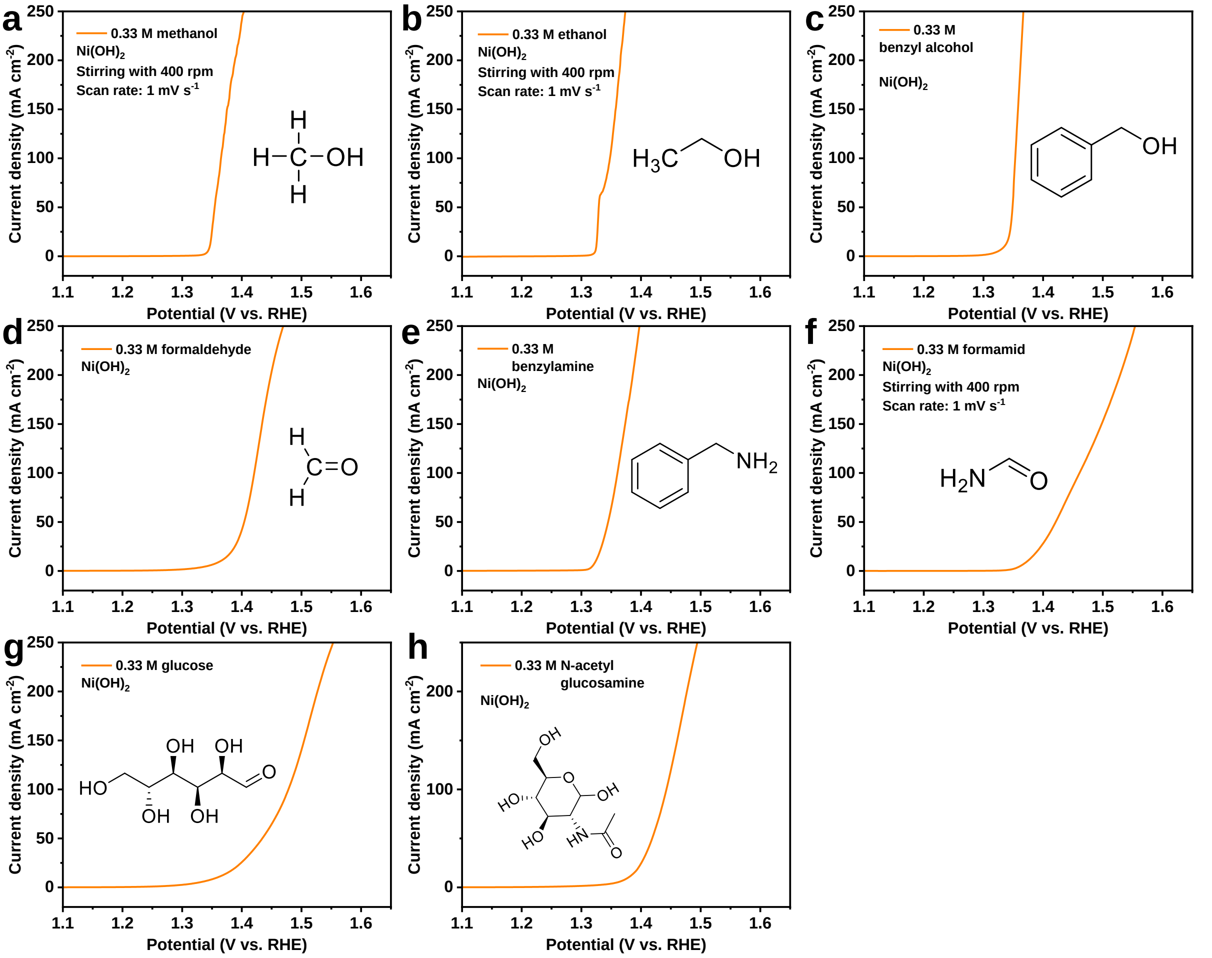

Supplementary Figure 18 | LSV polarization curves for $Ni(OH)_2$ electrode in 1 M KOH + 0.33 M organics. **a,** methanol, **b,** ethanol, **c,** benzyl alcohol, **d,** formaldehyde, **e,** benzylamine, **f,** formamide, **g,** glucose, and **h,** N-acetyl glucosamine.

The LSV polarization curves show that the Ni^{3+} is highly active for these selected organics, containing hydroxyls (methanol, ethanol, and benzyl alcohol), carbonyls (formamide, urea, formaldehyde, glucose, and N-acetyl glucosamine), and amino (benzylamine).

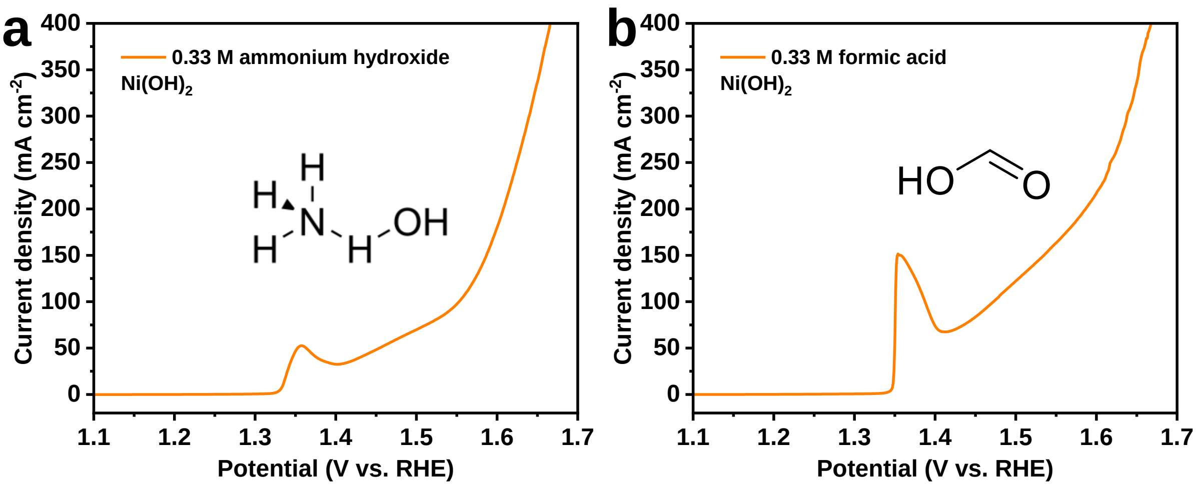

Supplementary Figure 19 | LSV polarization curves for $Ni(OH)_2$ electrode at 1 M KOH + 0.33 M organics. **a,** Ammonium hydroxide, and **b,** formic acid, respectively. The LSV polarization curves showed that the Ni^{3+} is low active for the ammonia and formic acid.

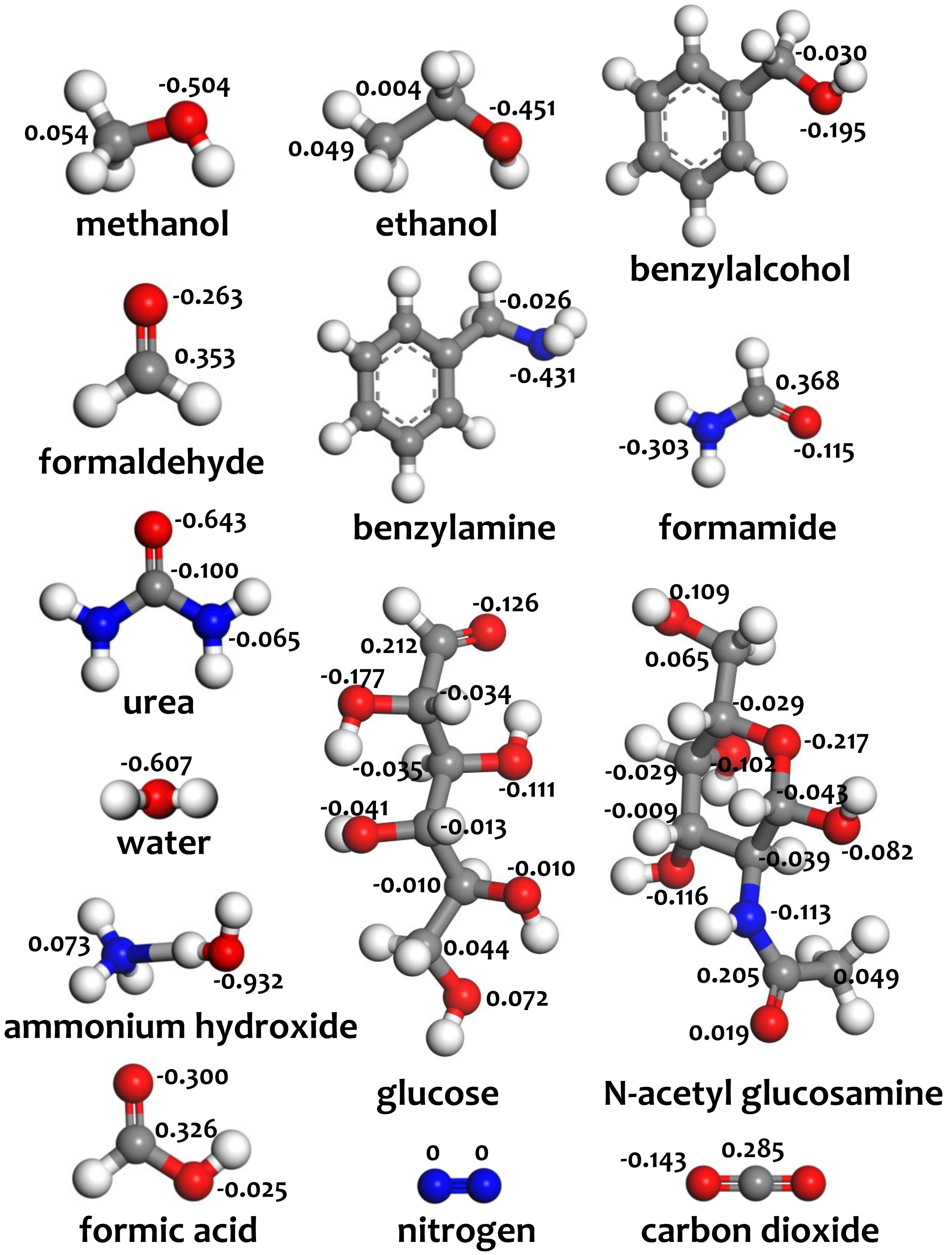

Supplementary Figure 20 | Molecular models for theoretical calculations, including HOMO level of organics and Δs_k values of nucleophilic atoms, in which the gray, red, blue, and white spheres are carbon, oxygen, nitrogen, and hydrogen respectively. The Δs_k values of the C, O, and N atoms in molecules are labeled.

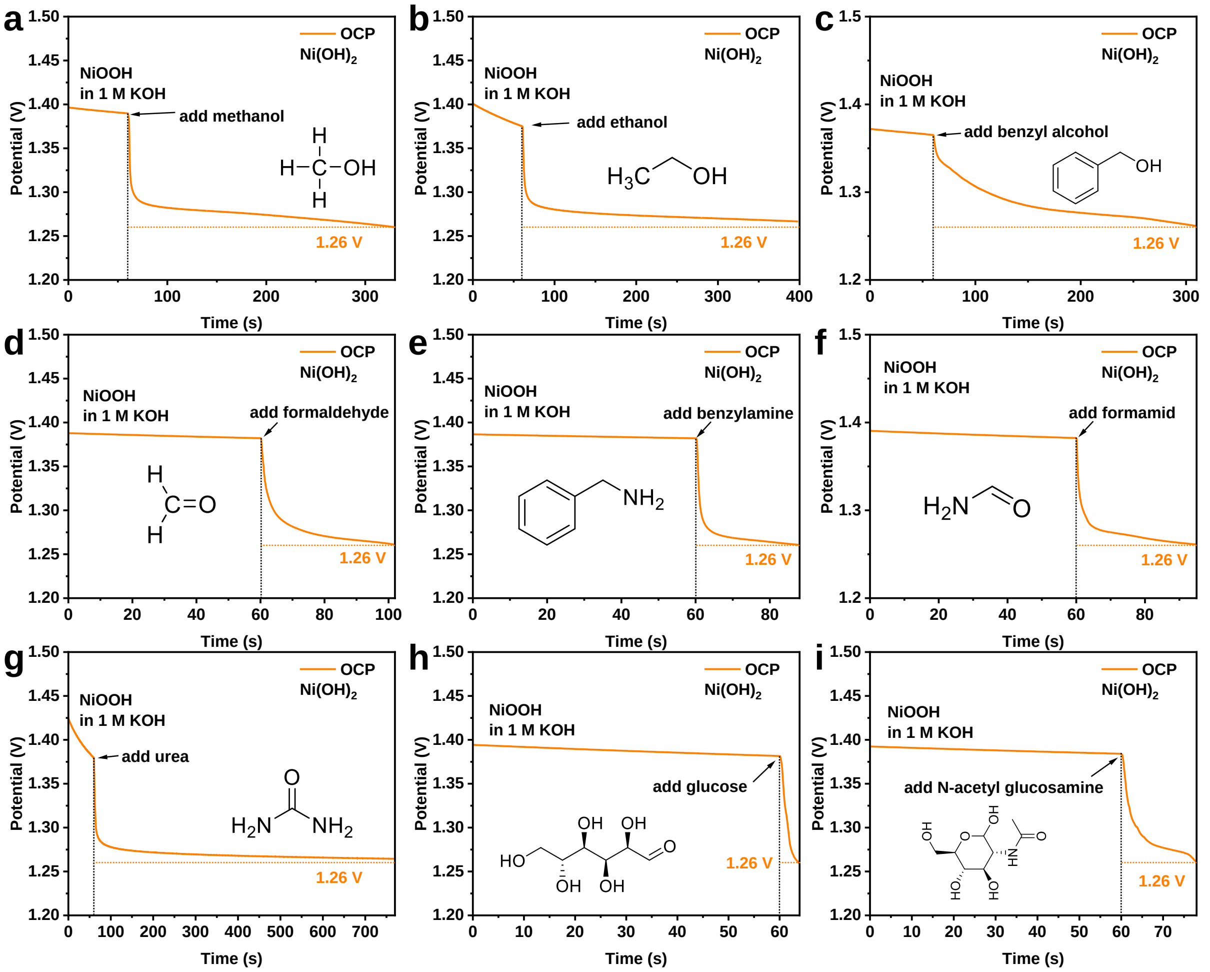

Supplementary Figure 21 | Open-circuit potential (V_{OCP}) decay monitored after Chronoamperometry measurement at 1.45 V to NiOOH steady state and then adding different organics containing 0.33 M **a,** methanol, **b,** ethanol, **c,** benzyl alcohol, **d,** formaldehyde, **e,** benzylamine, **f,** formamide, **g,** urea, **h,** glucose, and **i,** N-acetyl glucosamine, respectively.

After polarizing the $Ni(OH)_2$ to NiOOH, introducing the organics will induce the rapid V_{OCP} decay to a quasi-equilibrium potential of 1.26 V, a potential region for Ni^{2+} generation, clearly indicating that the Ni^{3+} oxidizing organics is a spontaneous chemical reaction.

Supplementary References

1 Kahn, A. Fermi level, work function and vacuum level. *Mater. Horizons* **3**, 7-10 (2016).

2 Görlin, M. *et al.* Tracking catalyst redox states and reaction dynamics in Ni-Fe oxyhydroxide oxygen evolution reaction electrocatalysts: the role of catalyst support and electrolyte pH. *J. Am. Chem. Soc.* **139**, 2070-2082 (2017).

3 Batchelor-McAuley, C. Defining the onset potential. *Curr. Opin. Electrochem.* **37**, 101176 (2023).
